# Musashi2 promotes EGF-induced EMT in pancreatic cancer via ZEB1-ERK/MAPK signaling

**DOI:** 10.1186/s13046-020-1521-4

**Published:** 2020-01-17

**Authors:** Weiwei Sheng, Xiaoyang Shi, Yiheng Lin, Jingtong Tang, Chao Jia, Rongxian Cao, Jian Sun, Guosen Wang, Lei Zhou, Ming Dong

**Affiliations:** 1 Department of Gastrointestinal Surgery, the First Hospital, China Medical University, Shenyang, 110001 China; 2 Department of General Surgery, the People’s Hospital of Liaoning province, Shenyang, 110034 China; 3 Department of General Surgery, the First Hospital of Nanchang University, NanChang, 330006 China; 4 Department of General Surgery, the Central Hospital of JingZhou City, JingZhou, 434020 China

**Keywords:** Musashi2, EGF, ZEB1, ERK/MAPK pathway, C-Myc, Epithelial mesenchymal transition, Pancreatic cancer

## Abstract

**Background:**

Our previous study showed Musashi2 (MSI2) promoted chemotherapy resistance and pernicious biology of pancreatic cancer (PC) by down-regulating Numb and p53. We further explored the novel molecular mechanism involving its oncogenic role in PC development.

**Methods:**

We investigated the potential role and mechanism of MSI2 in EGF-induced EMT in PC in vitro and vivo.

**Results:**

EGF enhanced EGFR (epidermal growth factor receptor) phosphorylation, induced EMT and activated ZEB1-ERK/MAPK signaling in 2 PC cells. However, MSI2 silencing reversed EGF stimulated function, including inhibiting EGF-promoted EMT-like cell morphology and EGF-enhanced cell invasion and migration. Meanwhile, MSI2 silencing inhibited EGF-enhanced EGFR phosphorylation at tyrosine 1068 and reversed EGF-induced change of the key proteins in EMT and ZEB1-ERK/MAPK signaling (ZEB1, E-cad, ZO-1, β-catenin, pERK and c-Myc). Additionally, MSI2 was co-stained and co-immunoprecipitated with ZEB1, pERK and c-Myc in PC cells by IF and co-IP, implying a close interaction between them. In vivo, MSI2 silencing inhibited pancreatic tumor size in situ and distant liver metastases. A close relationship of MSI2 with EMT and ZEB1-ERK/MAPK signaling were also observed in vivo and human PC samples, which coordinately promoted the poor prognosis of PC patients.

**Conclusions:**

MSI2 promotes EGF-induced EMT in PC via ZEB1-ERK/MAPK signaling.

## Background

Pancreatic cancer (PC) is one of the most malignant tumors in the world. From 2000 to 2011, the incidence rate and age-standardized mortality of PC in Chinese men have greatly increased, ranking the top first and second among malignant tumors [1]. In 2018, about 55,440 people were diagnosed with and about 44,340 died of PC in United States [2]. PC is now predicted to become the second cancer related death in 2020 [3]. Aggressive local invasion and distant metastase contribute to the poor prognosis of PC patients which is significantly promoted by epithelial-mesenchymal transition (EMT) [4]. PC stimulated by EMT loses epithelial character, gains strong invasive mesenchymal cells, and finally transforms into the highly malignant phenotype [2, 5].

Musashi RNA-binding protein 2 (MSI2) is a translational repressor identified in *Drosophila melanogaster*. It plays a significant role in regulating asymmetric division, stemness maintenance and neural differentiation, hematopoietic and gastrointestinal systems in many species [6–9]. Recently, MSI2 dysregulation has been reported in various hematology and solid tumors, including acute and chronic myeloid leukemia (AML and CML), brain, lung, gastric, hepatocellular and bladder cancers [10–15]. Our previous study first showed MSI2 overexpression promoted cell invasion and metastasis in PC by down-regulating Numb in vivo and vitro [16]. Additionally, under gemcitabine or cisplatin treatment, MSI2 promoted chemotherapy resistance and pernicious biology of PC in p53-dependent manner [17]. In current study, we intend to investigate the novel signaling involving the oncogenic role of MSI2 in advanced progression of PC. We first found MSI2 promoted EGF-induced EMT in PC through ZEB1-ERK/MAPK signaling in vitro and vivo, which has not been reported previously, to our knowledge.

## Materials and methods

### Tissue samples and cell lines

This study was approved by the academic review board of the first hospital of China Medical University and the approval in consent form that was signed by each resident patient. Seventy four paraffin-embedded ductal adenocarcinoma samples were obtained from postoperative patients at the First Hospital of China Medical University between 2010 and 2015 with complete follow up data. AsPC-1, BxPC-3, PANC-1, Miapaca-2 and SW1990 human PC cell lines were purchased from the Cell Bank of the Chinese Academy of Sciences (Shanghai, China). These cell lines were maintained in recommended growth media with 10% fetal calf serum (Gibco Invitrogen, Carlsbad, CA).

### Immunofluorescence (IF) staining

PC cells were plated into 24-well culture plates, fixed in 4% paraformal dehyde, permeabilized with 0.25% Triton X-100 and incubated with 5% BSA. Then the slices were stained with the primary antibodies: MSI2 (Abcam, Cambridge, UK), ZEB1 (Proteintech, Chicago, IL, USA), pERK (Cell signaling technology, MA, USA) and c-Myc (Proteintech). The secondary antibodies were conjugated with FITC and TRITC (Vector laboratories, California, USA). Cells were then co-stained with blue Hoechest33258 (Vector laboratories) for nuclear visualizing and detected under microscope (Nikon Microphot-FX, Japan).

### Immunohistochemistry

As described previously [18], 4-um sections were covered with 0.3% H_2_O_2_, subjected to high pressure and added with 10% normal goat serum. Then the slices were incubated with primary antibodies: MSI2 (Abcam), ZEB1 (Proteintech), E-cadherin(E-cad, Abcam), c-Myc (Proteintech), pERK (Cell signaling technology), ZO-1 (Proteintech), β-catenin (Proteintech) and Vimentin (Proteintech). After 3 times wash in PBS, slices were incubated with the secondary antibodies, treated with streptavidin–peroxidase reagent, visualized with DAB, counterstained with haematoxylin and detected under microscope. Staining intensity was scored as 0–3 (negative, weak, medium and strong). Extent of staining was scored as 0 (< 5%), 1 (5–25%), 2 (26–50%), 3 (51–75%), and 4 (> 75%) according to the positive staining areas to the whole carcinoma. The final scores were calculated by three pathologists. The same score evaluation was used in both human PC samples and in vivo.

### Western blot

Whole protein lysates were prepared from sg1-MSI2, sg2-MSI2, MSI2-GFP, MSI2-GFP plus MSI2siRNA and scramble transfected PC cell lines with or without EGF (50 ng/ml). Samples were loaded onto 10% SDS-polyacrylamide gels, transferred to PVDF membranes and incubated with primary antibodies: MSI2 (Abcam), ZEB1 (Proteintech), E-cad (Abcam), N-cadherin (N-cad, Abcam), c-Myc (Proteintech), pERK (Cell signaling technology), Cavelino-1 (Proteintech), ZO-1 (Proteintech), β-catenin (Proteintech), MMP9 (Abcam), Vimentin (Proteintech), alpha smooth muscle actin (a-SMA, Abcam), GAPDH (Proteintech), the phosphorylated epidermal growth factor receptor (EGFR) at tyrosine 845 (pEGFR845, Abcam), the phosphorylated site at tyrosine 992 (pEGFR992, Cell signaling technology) and at tyrosine 1068 (pEGFR1068, Abcam). Then, membranes were incubated with secondary antibodies (Proteintech). Protein bands were detected with an ECL detection kit (Thermol Biotech Inc., USA). Each experiment was repeated 3 times.

### Immunoprecipitation

As described previously [18], whole protein lysates prepared from AsPC-1 and BxPC-3 cells were extracted in the IP lysis buffer. Briefly, MSI2, ZEB1, c-Myc and pERK and IgG (Santa Cruz) antibodies were pre-incubated with magnetic beads (Bio-Rad, California, USA) at 4 °C. Then antibody-beads complex was washed 3 times and incubated with the supernatants of protein lysates overnight at 4 °C. The immunocomplexes were eluted by boiling in sample loading buffer for SDS–PAGE, and then subjected to WB analysis with MSI2, ZEB1, pERK and c-Myc antibodies. Each experiment was repeated 3 times.

### CRISPR/Cas9 and siRNA mediated MSI2 silencing and lentivirus vector mediated MSI2 overexpression

Lentivirus(Lenti)-cas9 and lenti-sgRNA were synthesized from Genechem (Genechem Co, Ltd., Shanghai, China). AsPC-1 and BxPC-3 cell lines were firstly transfected with lenti-cas9 and next selected by puromycin (Sigma). The stable sub-lines were then transfected with MSI2-sgRNA (sg1-MSI2 and sg2-MSI2) and sgRNA control (scramble) to specifically silence target genes. MSI2siRNA and siRNAcontrol were synthesized from GenePharma Company (GenePharma Co, Ltd). Cells were transiently transfected with siRNA via oligofectamine3000 (Invitrogen, Carlsbad, CA, USA) under the protocol. Lentivirus vector (GV358) mediated MSI2 overexpression (MSI2-GFP) and corresponding control vector (GFP) were also synthesized from Genechem. SW1990 cells with MSI2 low expression was used to construct MSI2 overexpressing stable PC cells following puromycin selection. The vector information and stable transfected efficiency (GFP fluorescence in MSI2 overexpressing stable SW1990 cells) were shown in Additional file 1: Figure S1. All target sequences mentioned above were shown in Additional file 2: Table S1.

### EMT construction

As our previous studies shown [5, 18], AsPC-1 and BxPC-3 cell transfected with sg1-MSI2, sg2-MSI2 and scramble were treated with 50 ng/ml EGF (Peprotech, RockyHill, New Jersey, USA) and 1%BSA (Sigma) triple times within 72 h, respectively. Cells were cultured with recommended growth media containing 0.5% FBS to enhance the efficiency of EGF. MSI-GFP and GFP transfected SW1990 cells were co-transfected with MSI2siRNA and treated with 50 ng/ml EGF mentioned above. The EMT construction was verified by the observation of EMT-like cell morphology (a spindle-shaped and fibroblast-like morphology), EMT-enhanced cell invasion and migration, and EMT-induced the change of EMT markers.

### Invasion and migration assays

Cell invasion were assessed with modified Boyden chamber (BD Biosciences, Sparks, MD, USA) assays. Briefly, sg1-MSI2, sg2-MSI2 and scramble transfected PC cells (pretreated with EGF for 48 h) was seeded onto 8.0-μM pore size membrane inserts coated with matrigel (BD Biosciences) in 24 well plates with FBS-free growth media plus EGF. Growth media plus 10% FBS was added to the bottom wells as a chemoattractant. Twenty four hours later, cells that had migrated to the underside of the inserts were stained with Crystal Violet Hydrate (Sigma, St. Louis, MO, USA). The migratory cells were counted in five random fields per well. Each experiment was repeated three times.

### In vivo xenograft model

Animals were maintained according to institutional regulations in facilities approved by the Animal Care Committee of China Medical University.

Total 20 nude mice (BALB/c-nu) were acclimatized for a week. Ten nude mice were used to construct pancreatic tumor in situ implanted with BxPC-3 cells (originated from primary PC). The other 10 nude mice were used to construct liver metastasis model implanted with AsPC-1 cells (originated from ascites of PC).

For pancreatic tumor in situ, a small horizontal laparotomy incision (1 cm) was made in left abdominal flank under chloral hydrate anesthesia. The spleen was identified and exposed. The injection site was the junction between spleen and pancreatic tail. sg-MSI2 and scramble transfected BxPC-3 cells (2 × 10^7^/ml) were suspended in pre-cold PBS (50ul) mixed with Matrigel (50ul) (BD Biosciences) and were then transplanted into tail of pancreas in10 nude mice. A cotton swab was used to avoid possible bleeding and leakage. The injection operation was shown in Additional file 3: Video S1.

The mice were sacrificed 1 month later. Tumors were resected and calculated using vernier calipers with the following formula: length x width x height × 0.52 in millimeters. Then, samples were extracted for late hematoxylin and eosin (HE) and IHC staining.

For liver metastasis model, as described in our previous studies [19], using the same incision mentioned above, the spleen was identified and exposed. A total of 1 × 10^7/ml sg-MSI2 and scramble transfected AsPC-1 cells suspended in pre-cold PBS (200ul) were injected into the lower-middle part of splenic capsule. A cotton swab was used to avoid bleeding and leakage from the injection site. The injection operation was shown in Additional file 4: Video S2. The mice were sacrificed 3 weeks later. The number of liver metastases was investigated immediately, and fixed for late HE and IHC staining.

### Statistical analysis

Using SPSS software 20.0 (SPSS, Chicago, IL, USA). The differences in WB, transwell assays, the volumes in orthotopic tumor and the numbers in liver metastases were expressed as mean ± *SE* and compared through Student’s *t*-test. The different proteins expression in vivo by IHC were compared through non-parametric test. The relationship between each target protein in human PC samples were analyzed by Spearman correlation tests. The Kaplan-Meier curve was used to estimate survival, and differences were analyzed by the log-rank test. A value of *P* < 0.05 indicated statistic significant.

## Results

### MSI2 location and the construction of MSI2 silencing stable cell lines

**Fig. 1:**
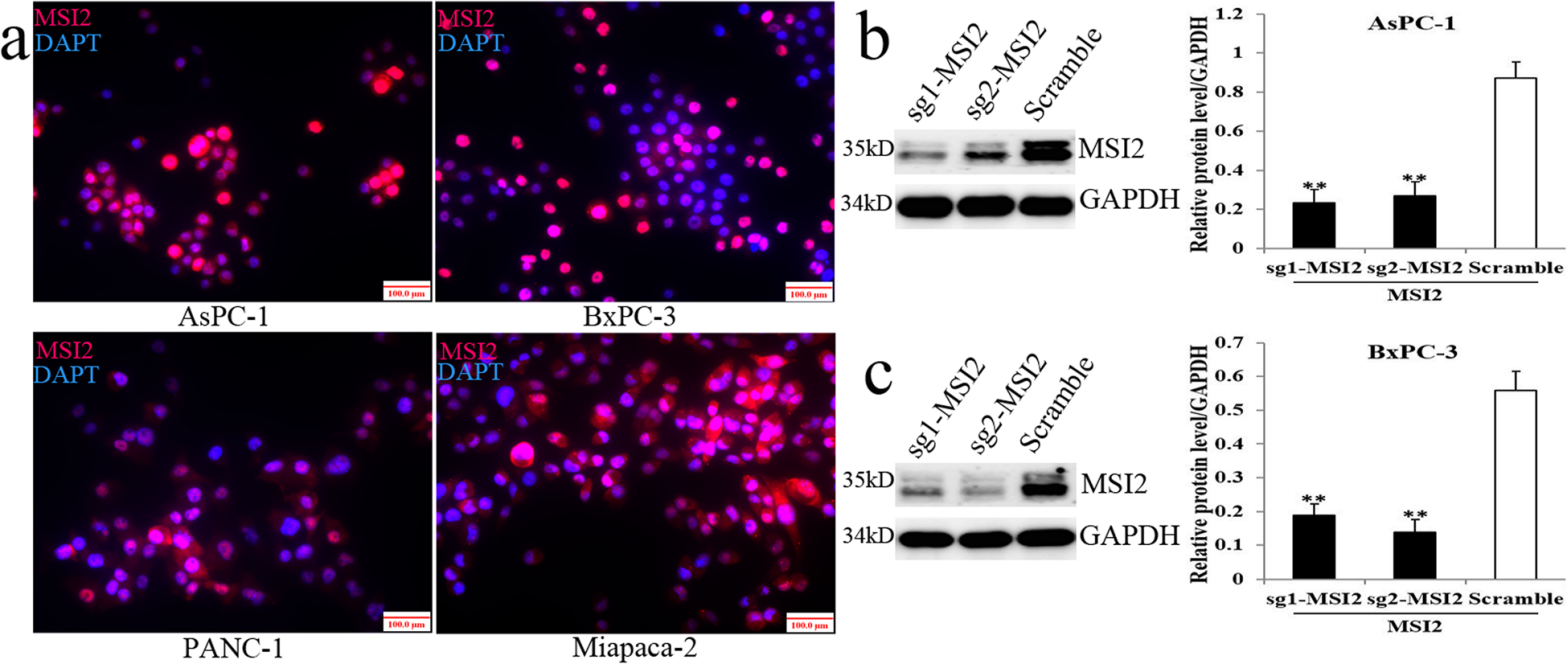
Nuclear and cytoplasmic location of MSI2 in PC cells by IF and the construction of MSI2 silencing stable PC cell lines via CRISPR/Cas9 system. **a** IF staining of MSI2 (TRITC, red) and nuclear (Hoechst, blue) in 4 PC cell lines (× 100 magnification). **b** and **c** MSI2 protein level in sg1-MSI2, sg2-MSI2 and scramble transfectedAsPC-1 (**b**) and BxPC-3 (**c**) cell lines detected by WB. The white bars: MSI2 protein expression in scramble groups. The black bars: MSI2 protein expression in sg1-MSI2 and sg2-MSI2 groups. **, *P* < 0.01 compared with the control

MSI2 showed predominantly nuclear and cytoplasmic expression in AsPC-1, BxPC-3, PANC-1 and Miapaca-2 cell lines by IF (Fig. 1a). Our previous studies showed EGF successfully induced EMT in MSI2 high expression AsPC-1 and BxPC-3 cell lines [5, 18]. Thus, CRISPR/Cas9 system mediated MSI2 silencing was constructed in above cell lines for the late EMT study. WB showed that MSI2 protein level in both 2 PC cell lines in sg1-MSI2 and sg2-MSI2 groups were significantly lower than that in corresponding scramble groups (Fig. 1b and c).

### MSI2 silencing inhibited EGF-induced EMT in 2 PC cell lines

**Fig. 2:**
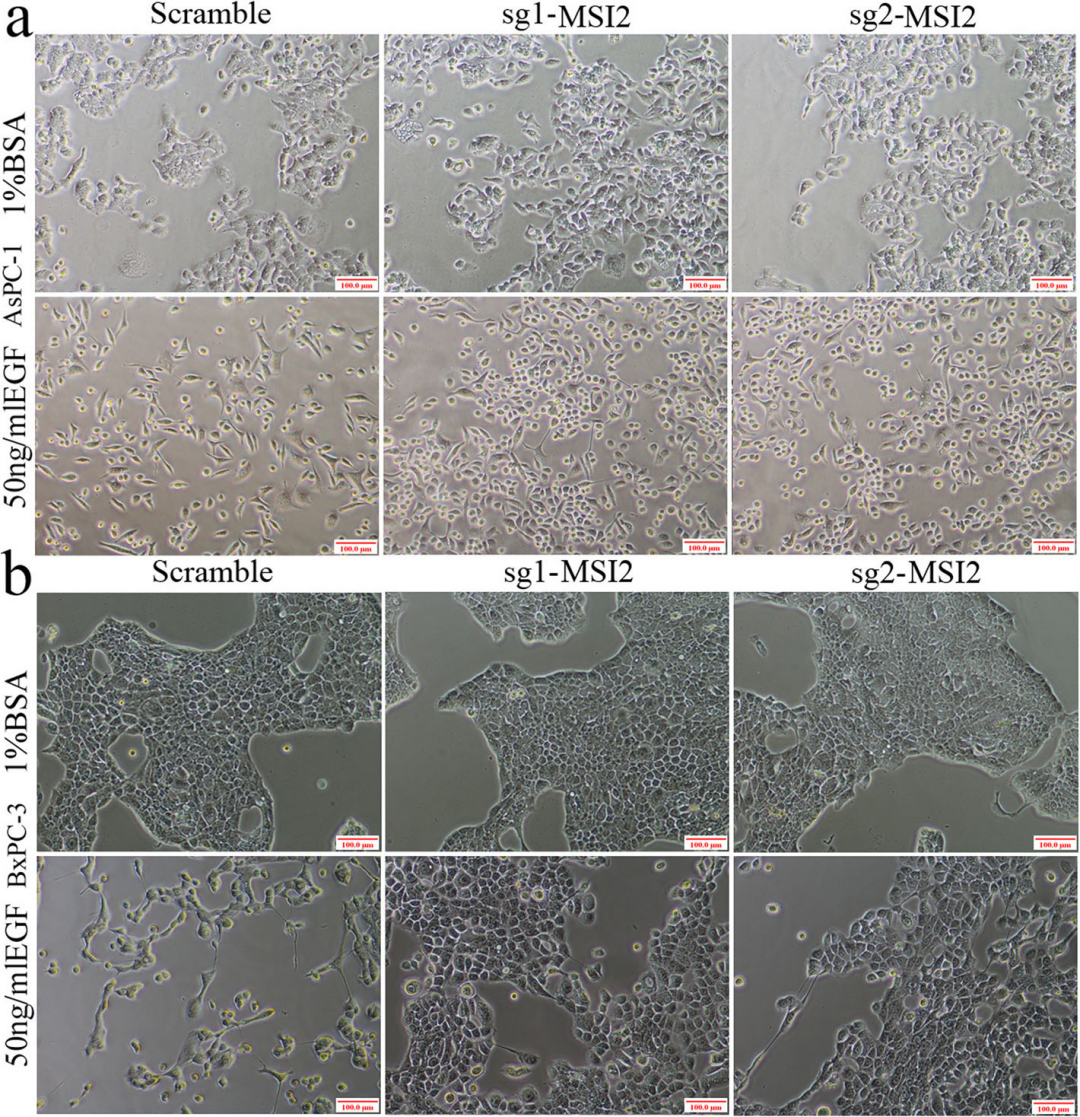
Cell morphology in sg1-MSI2, sg2-MSI2 and scramble transfected AsPC-1 and BxPC-3 cells with or without EGF (50 ng/ml) treatment. **a** and **b** Under EGF treatment, the fibroblastoid-like phenotype in AsPC-1 (**a**) and BxPC-3 (**b**) cells in scramble, sg1-MSI2 and sg2-MSI2 groups

Upon EGF, both AsPC-1 and BxPC-3 cells exhibited EMT-like cell morphology: cells lost their epithelial character, and exhibited a spindle-shaped and fibroblast-like morphology (Fig. 2). MSI2 silencing alone had no effect in cell morphology, but significantly inhibited EGF-induced EMT-like cell morphology. In sg1-MSI2 and sg2-MSI2 groups, EGF-treated AsPC-1 and BxPC-3 cells restored their original cell morphology with little spindle-shaped and fibroblast-like morphology, compared with scramble groups (Fig. 2).

**Fig. 3:**
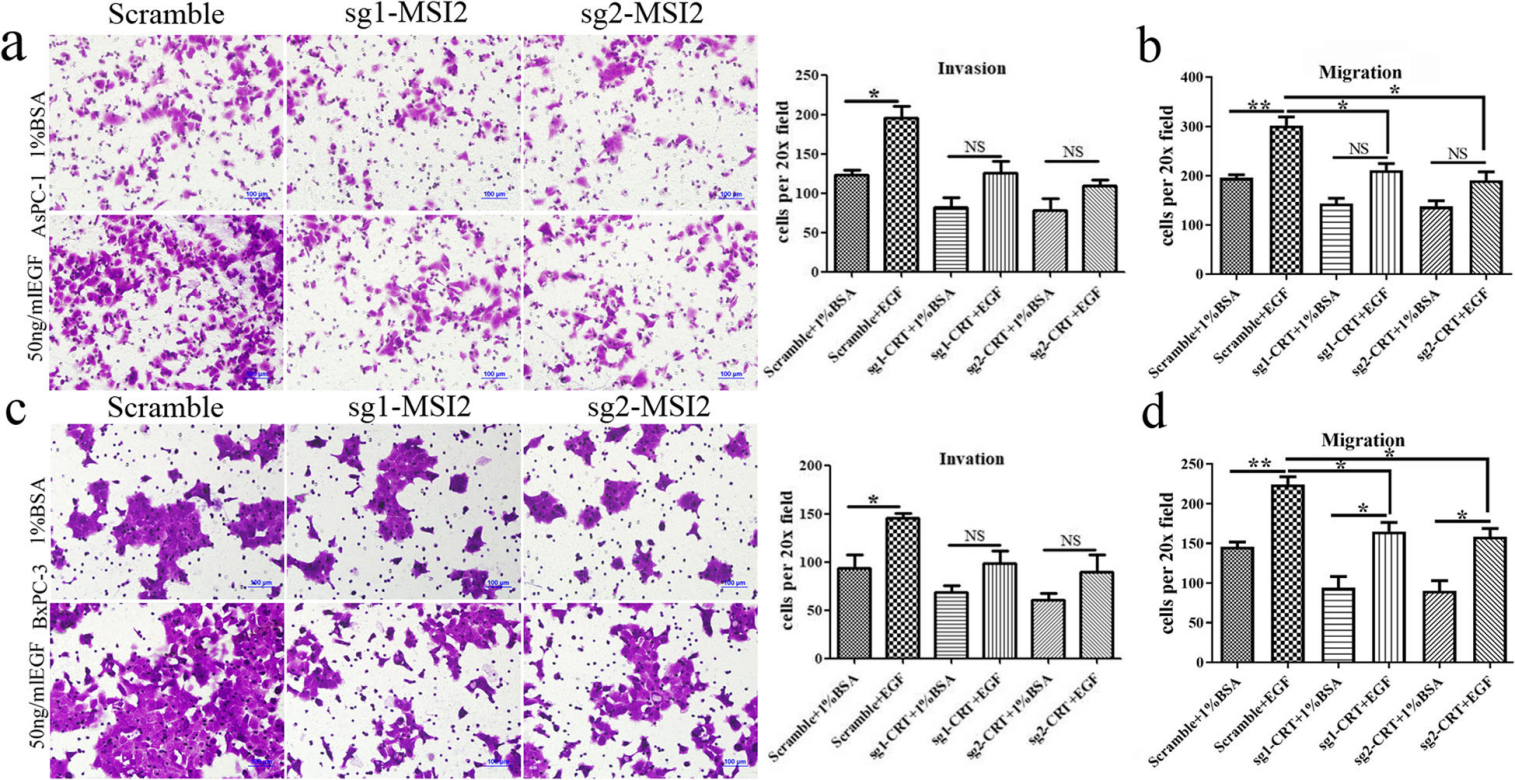
MSI2 silencing inhibited EGF-induced Cell invasion and migration in PC cells. **a** and **b** Cell invasion (**a**) and migration (**b**) in sg1-MSI2, sg2-MSI2 and scramble transfected AsPC-1 cells with or without EGF (50 ng/ml) treatment. **c** and **d** Cell invasion (**c**) and migration (**d**) in sg1-MSI2, sg2-MSI2 and scramble transfected BxPC-3 cells with or without EGF (50 ng/ml) treatment. Bars indicate ± S.E.*, *P* < 0.05; **, *P* < 0.01 compared with the control

The aggressiveness of PC cells is significantly driven by EMT [4]. We found that EGF significantly stimulated cell invasion and migration in both AsPC-1 and BxPC-3 cells (Fig. 3). MSI2 silencing alone without EGF partially inhibited cell invasion and migration. However, this trend is much more significant with EGF treatment. Upon EGF, a significant increase of cell invasion and migration were found in scramble groups compared with sg1-MSI2 and sg2-MSI2 groups. Namely, the growing difference of cell motility between scramble groups with and without EGF was much more obvious than that in sg-MSI2 groups (Fig. 3).

**Fig. 4:**
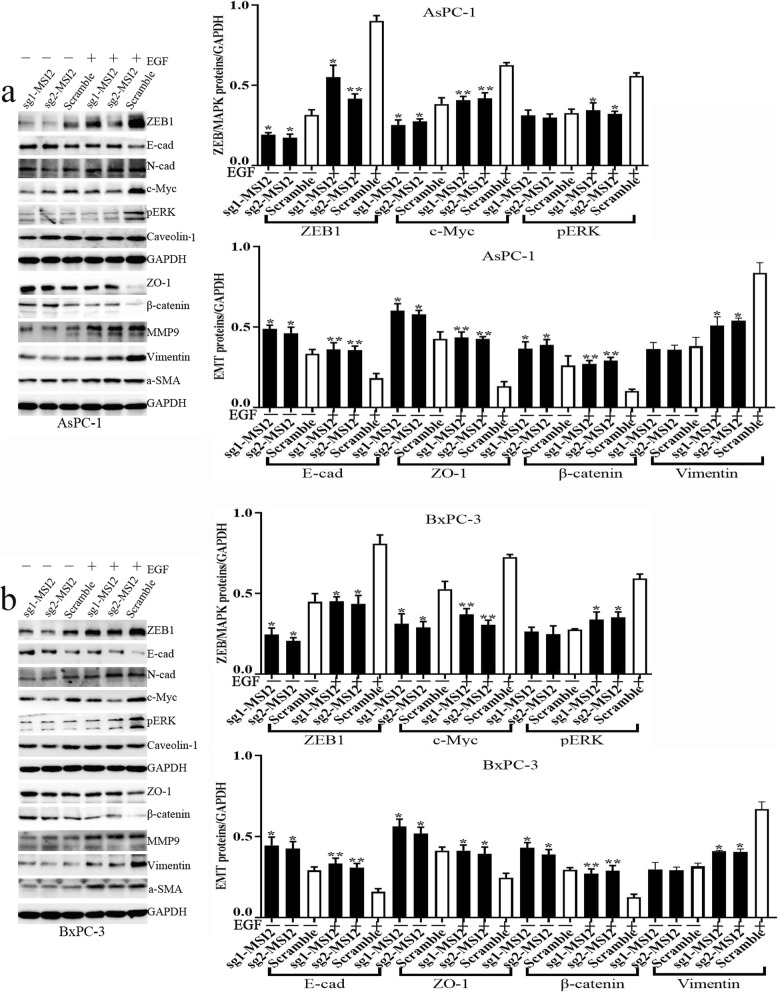
MSI2 silencing inhibited EGF-induced EMT and ZEB1-ERK/MAPK signaling. **a** and **b.** The protein expression involving EMT and ZEB1-ERK/MAPK signaling in sg1-MSI2, sg2-MSI2 and scramble transfected AsPC-1 (**a**) and BxPC-3 (**b**) cells with or without EGF (50 ng/ml) treatment. The white bars: target protein expression in scramble groups with or without EGF treatment. The black bars: target protein expression in sg1-MSI2 and sg2-MSI2 groups with or without EGF treatment. Bars indicate ± S.E.*, *P* < 0.05; **, *P* < 0.01 compared with the control

Meanwhile, EGF significantly induced the change of EMT epithelial and mesenchyme markers in PC cells. In detail, EGF induced decrease of epithelial markers E-cad, ZO-1 and β-catenin and increase of mesenchyme markers MMP9, vimentin and a-SMA in both AsPC-1 and BxPC-3 cells (Fig. 4). MSI2 silence alone without EGF stimulus partially upregulated E-cad, ZO-1 and β-catenin expression. This trend is much more significant upon EGF. Moreover, MSI2 silencing also inhibit EGF-induced increase of vimentin (Fig. 4).

Taken together, MSI2 silencing inhibited EGF induced EMT in vitro.

### MSI2 silencing inhibited EGF-stimulated ZEB1-ERK/MAPK signaling in 2 PC cells

**Fig. 5:**
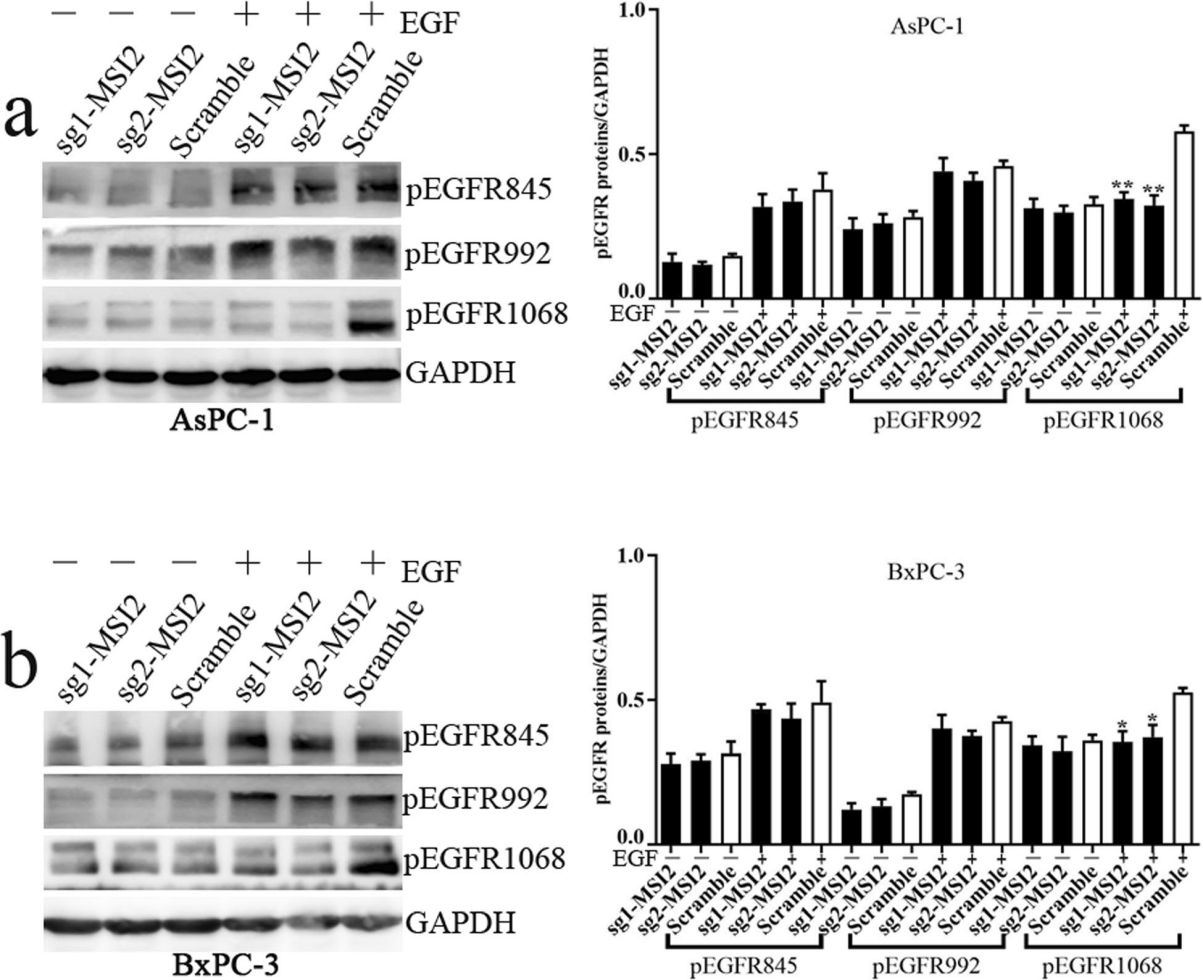
The effect of MSI2 silencing in EGF induced pEGFR expression. **a** and **b** The expression of pEGFR845, pEGFR992 and pEGFR1068 in sg1MSI2, sg2MSI2 and scramble transfected AsPC-1(**a**) and BxPC-3 cells (**b**) with or without EGF treatment

It is well known that EGF is the prototypic and founding member of the EGFR ligand family. EGF binding triggers receptor autophosphorylation on key cytoplasmic residues. The phosphorylated receptor activates complex downstream signaling cascades, including the ERK/MAPK pathway. Thus, we first detected the expression of phosphorylated EGFR at different sites of tyrosine in EGF treated PC cells upon EGF. In Fig. 5, EGF activated phosphorylation of EGFR at the sites of tyrosine 845 (pEGFR845), 992 (pEGFR992) and 1068 (pEGFR1068) in vitro. All their expression was significantly increased in EGF treated AsPC-1 and BxPC-3 cells. However, MSI2 silence specifically inhibited EGF activated pEGFR1068 in both cell lines. The expression of pEGFR845 and pEGFR992 were unchanged (Fig. 5). Thus, we inferred that MSI2, as an upstream regulator, specially regulated EGF activated pEGFR1068 and its downstream of ERK/MAPK signaling in PC cells.

ZEB1 is a key element of a network of transcription factors that controls EMT [20]. ERK/MAPK pathway and its key downstream target c-Myc plays a vital role in the initiation of EMT in normal epithelial and cancer cells [21–25]. Meanwhile, EMT in lung cancer cells was meditated by the close interaction between ERK and ZEB1 pathway [26]. Thus, we next investigated whether MSI2 mediated EGF induced EMT via ZEB1-ERK/MAPK signaling.

**Fig. 6:**
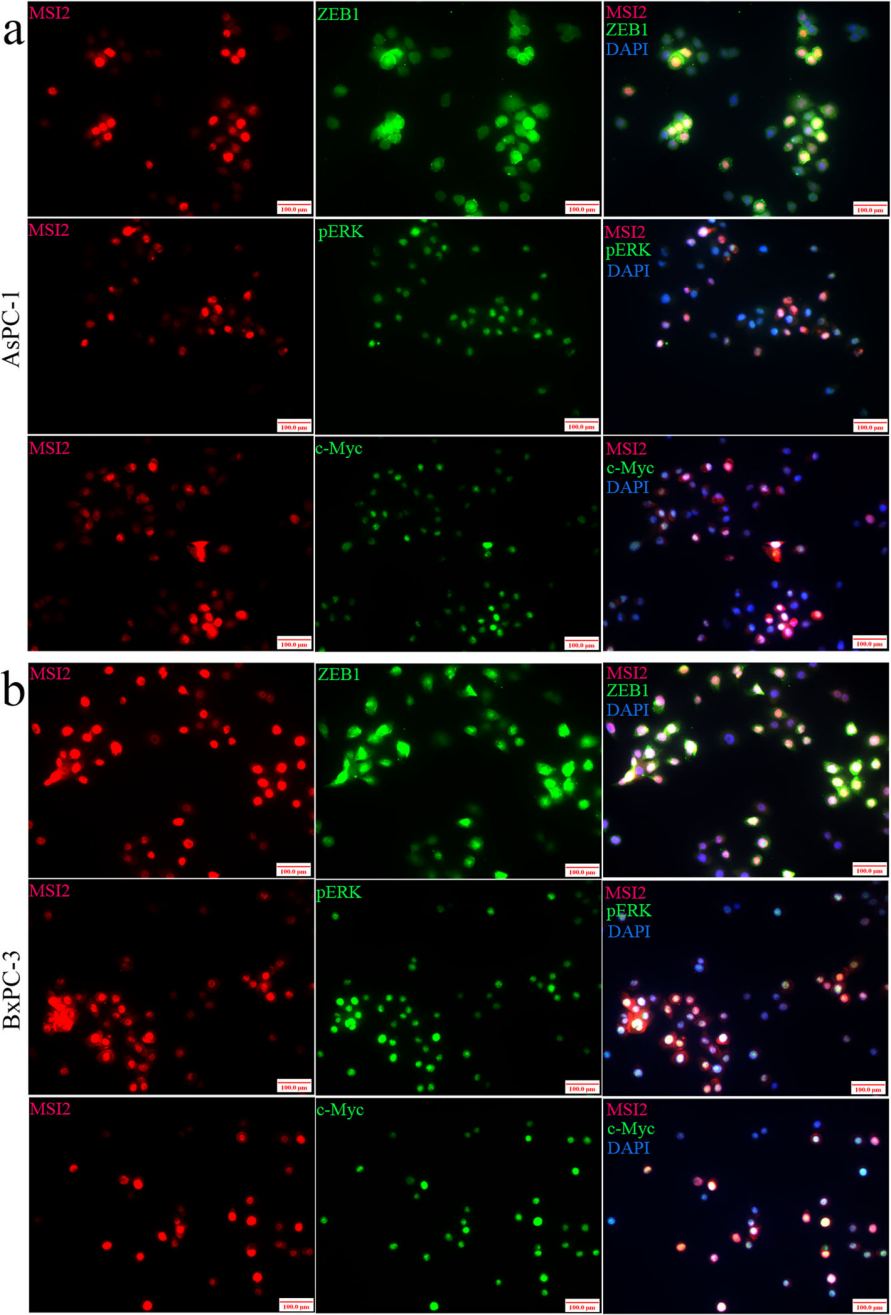
The co-staining of MSI2 with ZBE1, pERK and c-Myc in PC cells by IF. **a** and **b** MSI2 was co-stained with ZBE1, pERK and c-Myc in predominant nucleus in AsPC-1 (**a**) and BxPC-3 (**b**) cells (× 100 magnification)

**Fig. 7:**
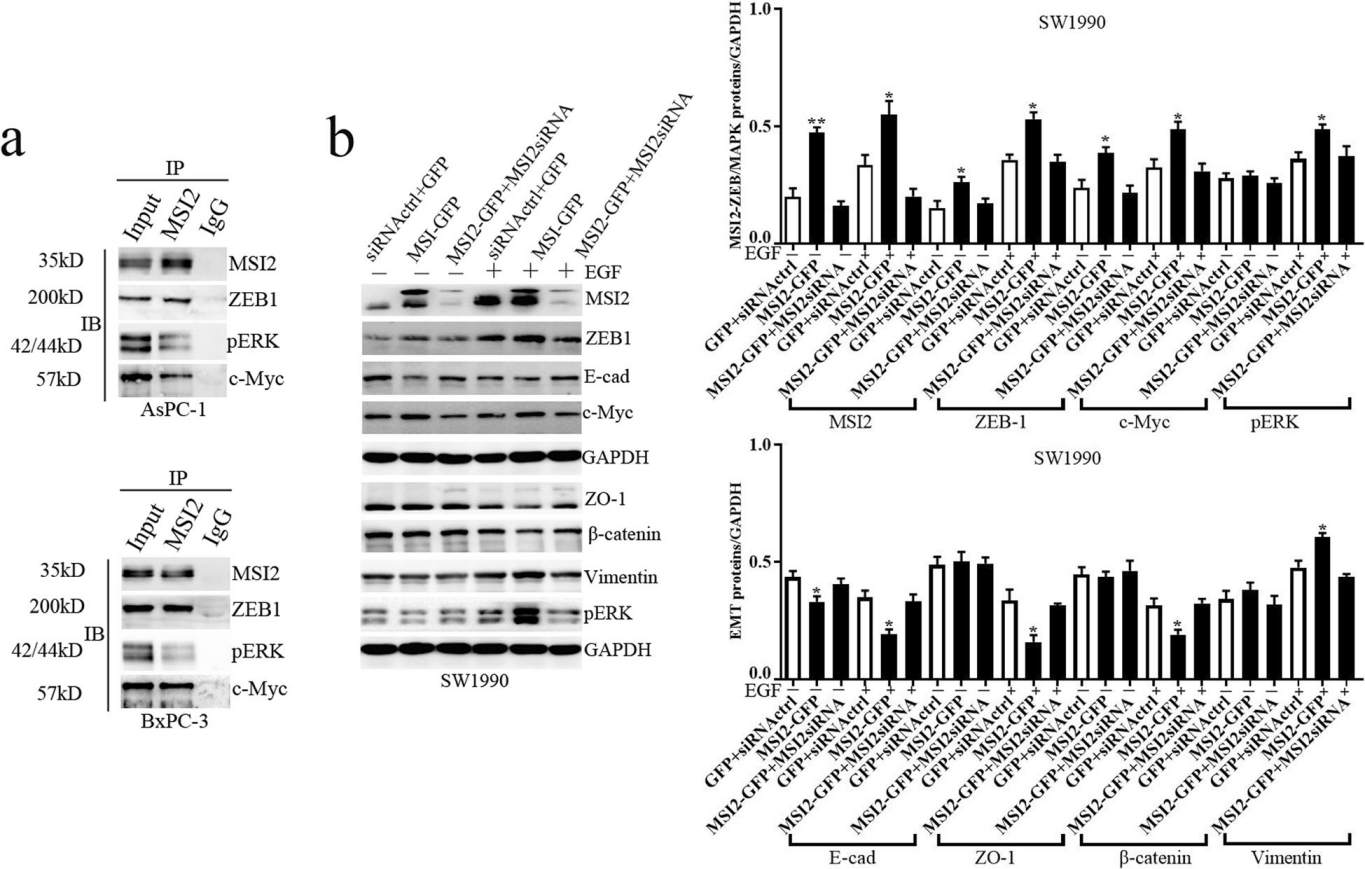
The interaction of MSI2 with EGF induced EMT and ZEB1-ERK/MAPK signaling was further verified by IP and rescue experiments. **a** AsPC-1 and BxPC-3 lysates were immunoprecipitated and WB. Input and IgG bands were used as positive and negative control, respectively. **b** MSI2 overexpression promoted EGF induced EMT and ZEB1-ERK/MAPK signaling which was inhibited by MSI2siRNA. Bars indicate ± S.E.*, *P* < 0.05; **, *P* < 0.01 compared with the control

In current study, EGF stimulated ZEB1-ERK/MAPK signaling in both AsPC-1 and BxPC-3 cells along with the increase of ZEB1, pERK and c-Myc protein expression (Fig. 4). Without EGF, MSI2 silence alone partially inhibited ZEB1 and c-Myc expression. Upon EGF, a significant decrease of ZEB1, pERK and c-Myc were observed in sgMSI2 and sgMSI2 groups compared with the corresponding scramble groups (Fig. 4). Meanwhile, MSI2/ZEB1, MSI2/pERK and MSI2/c-Myc were co-stained in predominant nuclear in normal AsPC-1 and BxPC-3 cells by IF (Fig. 6). Moreover, MSI2 was co-immunoprecipitated with above proteins in the lysates in both cell lines (Fig. 7a), implying a close interaction between MSI2 and ZEB1-ERK/MAPK signaling pathway.

For the rescue experiment, SW1990 PC cells with low MSI2 expression were firstly used to construct MSI2 overexpression stable cell line (MSI2-GFP). WB showed that MSI2 expression was significantly higher in MSI2-GFP group than that in scramble GFP group whatever with or without EGF treatment (Fig. 7b). Meanwhile, MSI2 overexpression in MSI2-GFP group was significantly inhibited when co-transfected with MSI2siRNA (Fig. 7b). Without EGF, MSI2 overexpression upregulated ZEB1 and c-Myc and downregulated E-cad expression, which was rescued by MSI2siRNA. Upon EGF, MSI2 overexpression promoted EGF-induced decrease of E-cad, ZO-1 and β-catenin and enhanced EGF-induced increase of c-Myc, pERK and Vimentin, all which were reversed by MSI2siRNA. Taken together with CRISPR/Cas9 mediated MSI2 silencing experiments, we conclude that MSI2 has a specific regulation in EGF-induced EMT in PC cells via ZEB1-ERK/MAPK signaling.

### MSI2 silencing inhibited pancreatic tumors in situ and liver metastasis in vivo

BxPC-3 and AsPC-1 cells were used to construct the models of pancreatic tumor in situ and distal liver metastasis, respectively.

**Fig. 8:**
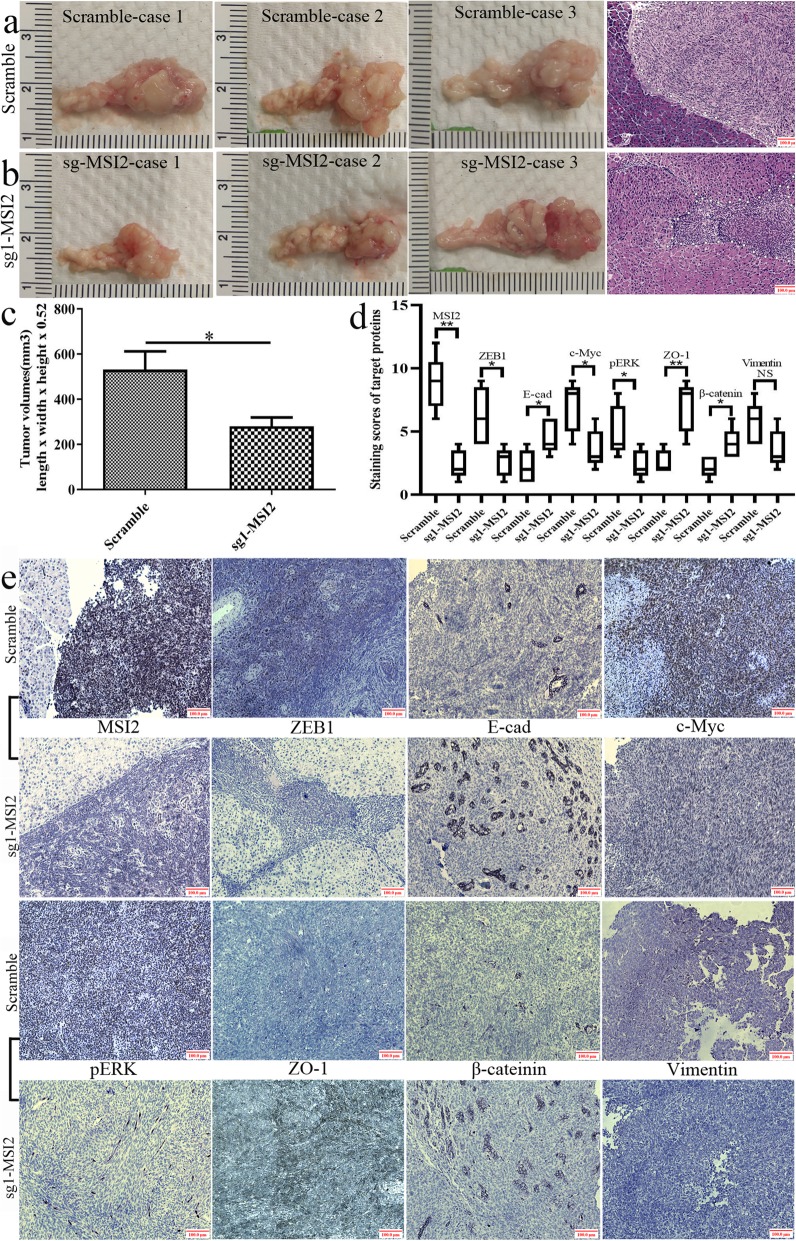
MSI2 silencing inhibited the growth of pancreatic tumors in situ. **a** Pancreatic tumors and corresponding HE staining (× 100 magnification) in scramble groups. **b** Pancreatic tumors and corresponding HE staining (× 100 magnification) in sg1-MSI2 groups. **c** The statistic analysis of tumor volume between scramble and sg1-MSI2 groups. **d** and **e** The statistic data (**d**) and representative images (**e**) in IHC assays of target proteins expression in vivo in sg1-MSI2 and scramble groups. Bars indicate ± S.E.*, *P* < 0.05; **, *P* < 0.01 compared with the control

Tumor volumes in the pancreas in sg1-MSI2 group were much smaller than that in paired corresponding scramble groups (*P* = 0.031) (Fig. 8a-c). HE staining also showed a large tumor area in scramble group compared with that in sg1-MSI2 groups (Fig. 8a, b). IHC further verified that MSI2, ZEB1, c-Myc and pERK expression were decreased (Fig. 8d, e) but E-cad, ZO-1 and β-catenin were increased in sg1-MSI2 groups compared with that in scramble groups (Fig. 8d, e). Vimentin showed high expression in scramble group, but no significant difference with sg1-MSI2 group.

**Fig. 9:**
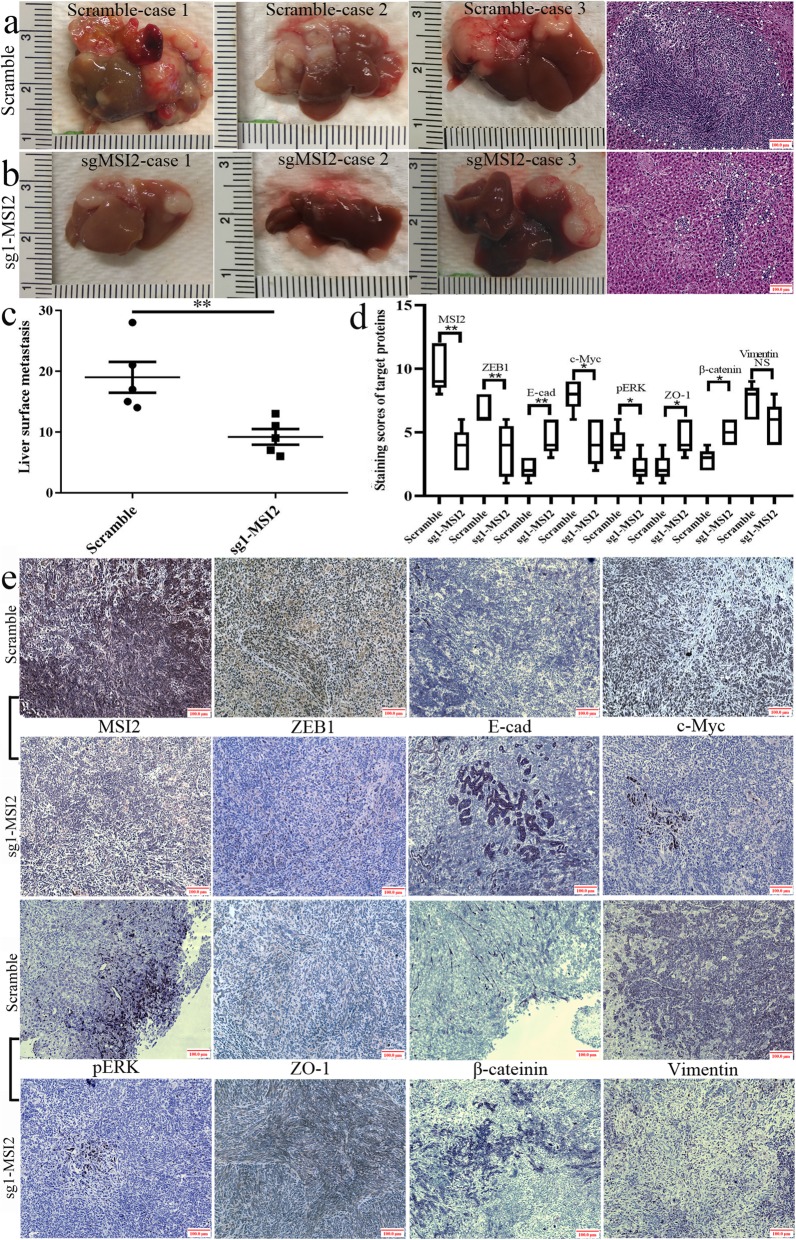
MSI2 silencing inhibited liver metastases in vivo. **a** Liver metastases and corresponding HE staining (× 100 magnification) in scramble groups. **b** Liver metastases and corresponding HE staining (× 100 magnification) in sg1-MSI2 groups. **c** The statistic analysis of liver metastasis number between scramble and sg1-MSI2 groups. **d** and **e** The statistic data (**d**) and representative images (**e**) in IHC assays of target proteins expression in vivo in sg1-MSI2 and scramble groups. Bars indicate ± S.E.*, *P* < 0.05; **, *P* < 0.01 compared with the control

The liver metastases in sg1-MSI2 group was much lower than that in scramble groups (*P* = 0.009) (Fig. 9a-c). HE staining also showed a large and serial area of liver metastasis in scramble group compared with that in sg-MSI2 groups only with scattered dot diffusion (Fig. 9a, b). IHC further verified that MSI2, ZEB1, c-Myc and pERK expression were increased, but E-cad, ZO-1 and β-catenin expression were decreased in sg1-MSI2 compared with the scramble groups (Fig. 9d, e). Vimentin showed high expression in scramble group, but no significant difference with sg1-MSI2 group.

### Clinical significance and the relationship of MSI2 with EMT and ZEB1-ERK/MAPK signaling in human PC samples

Finally, we investigated the close relationship between MSI2, ZEB1 (the key regulator of EMT), c-Myc (the key target of ERK/MAPK signaling), E-cad (EMT epithelial marker) and vimentin (EMT mesenchyme markers) with the clinical significance of PC patients.

The location of MSI2, ZEB1 and c-Myc in nucleus sand cytoplasm, and vimentin in membrane and cytoplasm were considered for scoring. E-cad membrane expression was identified as normal expression, whereas its negative or cytoplasmic expression were identified as abnormal expression. In 74 PC samples, proteins were overexpressed in 67.6% (50/74) of MSI2, 52.7% (39/74) of ZEB1, 39.2% (29/74) of E-cad, 51.3% (38/74) of c-Myc and 29.7% (22/74) of vimentin by IHC assays, respectively (Table 1).

**Table 1:** The relationship of MSI2 with ZEB1, E-cad, Vimentin and c-Myc expression in clinical samples

Parameters		CRT		Spearman ranks	*P*
		Low (24)	High (50)		
ZEB1	Negative (35)	16	19	0.269	0.021
	Positive (39)	8	31		
E-cad	Abnormal (45)	10	35	−0.272	0.019
	Normal (29)	14	15		
c-Myc	Negative (36)	16	20	0.250	0.032
	Positive (38)	8	30		
Vimentin	Negative (52)	20	32	0.198	0.089
	Positive (22)	4	18		

**Fig. 10:**
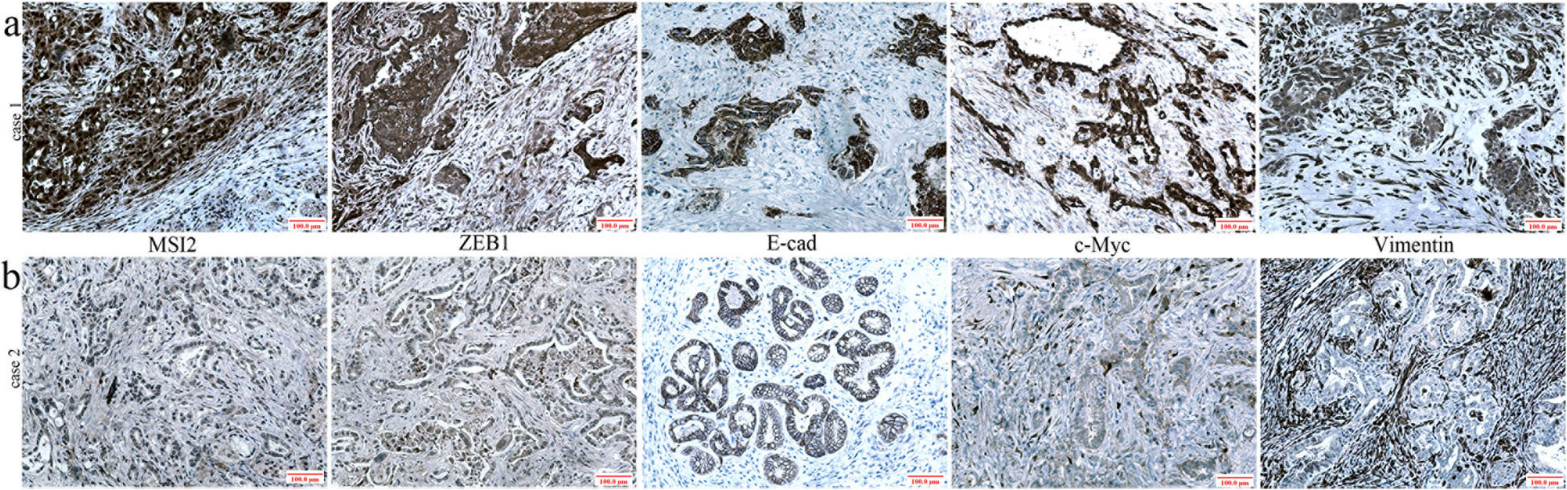
MSI2, ZEB1, c-Myc, pERK, E-cad and Vimentin expression in human PC samples. **a** High expression of MSI2, ZEB1,c-Myc and Vimentin and abnormal expression of E-cad in one case of PC tissues. **b** Weak expression of MSI2, ZEB1, c-Myc and Vimentin and normal expression of E-cad in another one case of PC tissues

MSI2 was positively associated with ZEB1 and c-Myc expression (*P* = 0.021 and *P* = 0.032) and negatively associated with normal E-cad expression (*P* = 0.019), but had no relationship with vimentin. In serial sections, PC tissues with high MSI2 expression were associated with positive ZEB1, c-Myc and abnormal E-cad expression (Fig. 10a), and vice versa (Fig. 10b). No association was found between MSI2 and vimentin, though their co-expression was showed in most PC tissues. These results were consistent with our observation in vitro and vivo.

**Fig. 11:**
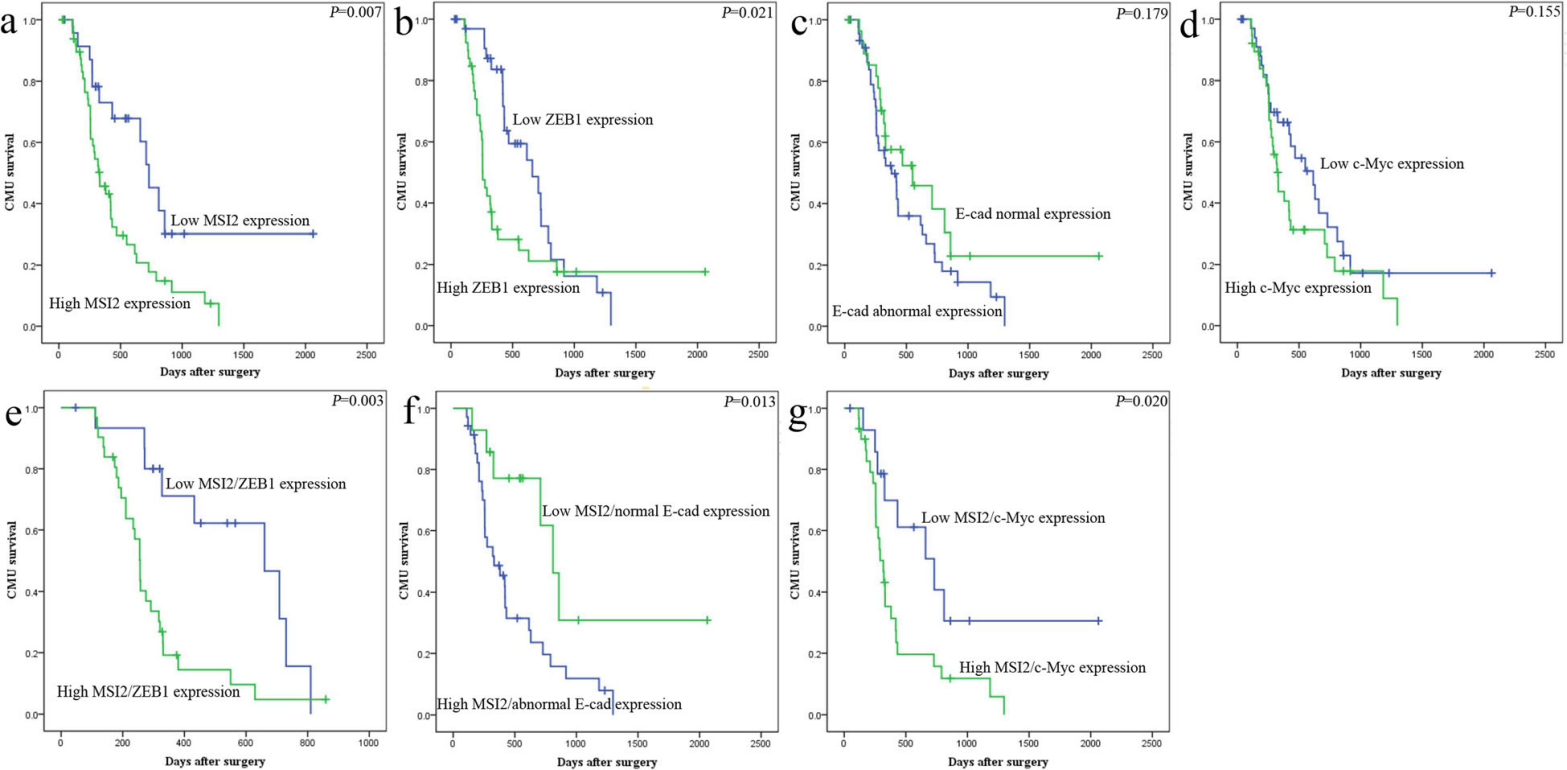
The relationship of MSI2, ZEB1, c-Myc, pERK and E-cad with the survival of 74 postoperative PC patients in Kaplan-Meier analysis. **a** High and low expression of MSI2 was plotted against overall survival time. **b** High and low expression of ZEB1 was plotted against overall survival time. **c** Normal and abnormal expression of E-cad was plotted against overall survival time. **d** High and low expression of c-Myc was plotted against overall survival time. **e**, **f** and **g** Co-expression of MSI2/ZEB1 (**e**), MSI2/E-cad (**f**) and MSI2/c-Myc (**g**) was plotted against overall survival time

The clinical significance of above protein in PC patients are well studied in our previous study and other reports [16, 17, 27–29]. Briefly, abnormal expression of above proteins was associated with multiple advanced clinical characters in PC patients. In current study, we concentrated on the relationship of their coordinate expression with the prognosis of PC patients. High expression of MSI2 and ZEB1 were associated with the poor prognosis of PC patients, respectively (*P* = 0.007 and *P* = 0.021) (Fig. 11a, b). Moreover, patients with the co-expression of MSI2 and ZEB1 had a much worse prognosis than patients with their low expression (*P* = 0.003) (Fig. 11e). Though E-cad and c-Myc had no relationship with the survival (Fig. 10c, d), patients combining with high MSI2 and abnormal E-cad expression or high MSI2 and c-Myc expression were associated with the worse prognosis (*P* = 0.013 and *P* = 0.020) (Fig. 10f, g). Taken together, co-expression of above proteins coordinately promoted the aggressive stage and poor prognosis of PC patients.

## Discussion

Accumulating studies have elucidated the frequency and mechanisms of MSI2 in multiple human hematology and solid cancers. For example, MSI2 silencing in AML cells increases sensitivity toward daunorubicin treatment via downregulating BCL2 and upregulating BAX [30]. MSI2 overexpression in a murine model of CML leads to downregulation of Numb by binding to the mRNA and preventing its translation [11]. MSI2 promotes the stemness and chemoresistance in liver cancer stem cells via LIN28A activation [31]. MSI2 promotes cell growth in breast cancer via increasing estrogen receptor 1 protein stability [32]. Our previous study also showed MSI2 promoted cell invasion and metastasis of PC by down-regulating Numb and enhanced drug resistance in p53-dependent manner [15, 16]. In current study, MSI2 promoted EGF-induced EMT in PC via ZEB1-ERK/MAPK signaling in vitro and vivo, which is a novel signaling in oncogenesis, to our knowledge.

We first found MSI2 silencing inhibited EGF-induced EMT in 2 PC cells, including inhibiting EGF-induced EMT-like cell morphology, EGF-enhanced cell invasion and migration, and EGF-stimulated change of EMT epithelia and mesenchyme markers. Similarly, MSI2 silencing represses extrahepatic cholangiocarcinoma growth and invasion by inhibiting EMT [33]. MSI2 silencing impairs cell proliferation and EMT in esophageal squamous cell carcinoma [34]. MSI2 depletion significantly down-regulates EMT markers (TGF-β receptor 1, SMAD3, SNAI1 and SNAI2) to inhibit non-small cell lung cancer metastasis [35]. Our study offers a new view involving the close interaction of MSI2 with EGF-induced EMT in PC, which has not been reported to our knowledge.

It is well known that EGFR plays a significant role in a number of varied processes in cancer progression such as cell adhesion, cell motility and invasion which are major steps in EMT event. Thus, we first detected if MSI2 regulated EGF induced EGFR phosphorylation in EMT inducing progression. We first found that MSI2, as an upstream regulator, specially regulated EGF activated pEGFR1068 and its downstream of ERK/MAPK signaling in PC cells.

ZEB1, as a transcription factor, promotes tumor invasion and metastasis by inducing EMT in many cancers [36–39]. ZEB1 recruiting multiple chromatin enzymes of E-cad promoter, which is the key mechanism in regulating EMT [40]. ERK/MAPK pathway and its major target downstream c-Myc were also indispensable in EMT development in various mammary epithelial and cancer cells [21–25, 41, 42]. Both c-Myc and MEK1-induced ERK2 nucleus localization are required for TGF-β-induced EMT in prostate cancer [25]. Additionally, the ERK-ZEB1 pathway mediates EMT in pemetrexed resistant lung cancer cells [26]. Loss of E-cad stimulates EGFR-MEK/ERK signaling, which promotes invasion via the ZEB1/MMP2 axis in non-small cell lung cancer [43]. Based on above studies, we further investigated whether MSI2 mediated EGF induced EMT via ZEB1-ERK/MAPK signaling. In current study, EGF significantly activated ZEB1-ERK/MAPK signaling following with the increase of ZEB1, pERK and c-Myc protein expression, which was significantly inhibited by MSI2 silencing. Meanwhile, MSI2 was co-stained and co-immunoprecipitated with ZBE1, pERK and c-Myc by IF and co-IP in both 2 PC cells. Moreover, further rescue experiments were verified above results. MSI2 overexpression promoted EGF induced EMT and ZEB1-ERK/MAPK signaling in vitro that was significantly inhibited by MSI2siRNA. In summary, with EGF stimulus, MSI2 interacted with ZEB1, upregulated ZEB1 and subsequently downregulated E-cad protein expression. Meanwhile, MSI2 promoted EGF-induced EGFR phosphorylation and EGF-activated ERK/MAPK signaling via interacted with the downstream target c-Myc. Finally, both above signaling pathways coordinately promoted EGF-induced EMT in PC cells mentioned above.

In vivo, MSI2 silencing inhibited pancreatic tumors in situ and liver metastasis, which was consistent with the studies in lung and esophageal squamous cell carcinoma [34, 44]. Moreover, in parallel with the results in vitro, the close relationship between MSI2 with EMT and ZEB1-ERK/MAPK signaling was also observed in vivo and in human PC samples. Meanwhile, co-expression of above target proteins was closely associated with the poor prognosis of PC patients. Taken together, MSI2 and ZEB1-ERK/MAPK signaling cooperatively contributed to the advance development of PC.

## Conclusion

In conclusion, MSI2 promotes EGF-induced EMT in PC cells via ZEB1-ERK/MAPK signaling pathway. Recently, accumulating studies focused on the function of the alternately spliced isoforms in MSI protein family. Upregulation of MSI2 variant 2 was observed in a large number of cancers by TCGA RNA-seq data sets [45]. Meanwhile, the small-molecule targeting of MSI2 activity is used in murine AML leukemia model [46]. The potential role of MSI2 isoforms and their small-molecule targets in PC will be further investigated in our future study.

## Supplementary information

**Additional file 1: Figure S1** The GV358 lentivirus vector information (a) and transfected efficiency (GFP fluorescence) in MSI2 overexpressing SW1990 cells (b).

**Additional file 2:** **Table S1**. The target sequences of sg1-MSI2, sg2-CRT, MSI2siRNA and corresponding scramble.

**Additional file 3.** The video of pancreas injection operation.

**Additional file 4.** The video of spleen injection operation.

## Data Availability

Materials are available upon request.

## Abbreviations

AML: Acute chronic myeloid leukemia
CML: Chronic myeloid leukemia
EGF: Epidermal growth factor
EGFR: Epidermal growth factor receptor
EMT: Epithelial mesenchymal transition
HE: Hematoxylin and eosin staining
IF: Immunofluorescence
IHC: Immunohistochemistry
IP: Immunoprecipitation
MSI2: Musashi-2
PC: Pancreatic cancer
WB: Western Blot
