# Correction to: Musashi2 promotes EGF-induced EMT in pancreatic cancer via ZEB1-ERK/MAPK signaling

**DOI:** 10.1186/s13046-020-01663-2

**Published:** 2020-08-24

**Authors:** Weiwei Sheng, Xiaoyang Shi, Yiheng Lin, Jingtong Tang, Chao Jia, Rongxian Cao, Jian Sun, Guosen Wang, Lei Zhou, Ming Dong

**Affiliations:** 1 Department of Gastrointestinal Surgery, the First Hospital, China Medical University, Shenyang, 110001 China; 2 Department of General Surgery, the People’s Hospital of Liaoning province, Shenyang, 110034 China; 3 Department of General Surgery, the First Hospital of Nanchang University, NanChang, 330006 China; 4 Department of General Surgery, the Central Hospital of JingZhou City, JingZhou, 434020 China

**Correction to: J Exp Clin Cancer Res 39, 16 (2020)**

**https://doi.org/10.1186/s13046-020-1521-4**

Following publication of the original article [1], the authors identified some minor errors in image-typesetting in Figs. 2 and 3; specifically in Fig. 2a, and Fig. 3b and Fig. 3d (corrected labels beneath the quantitative histograms). The correct figure is given below. All correction had no any effect to the final conclusion.

**Fig. 2:**
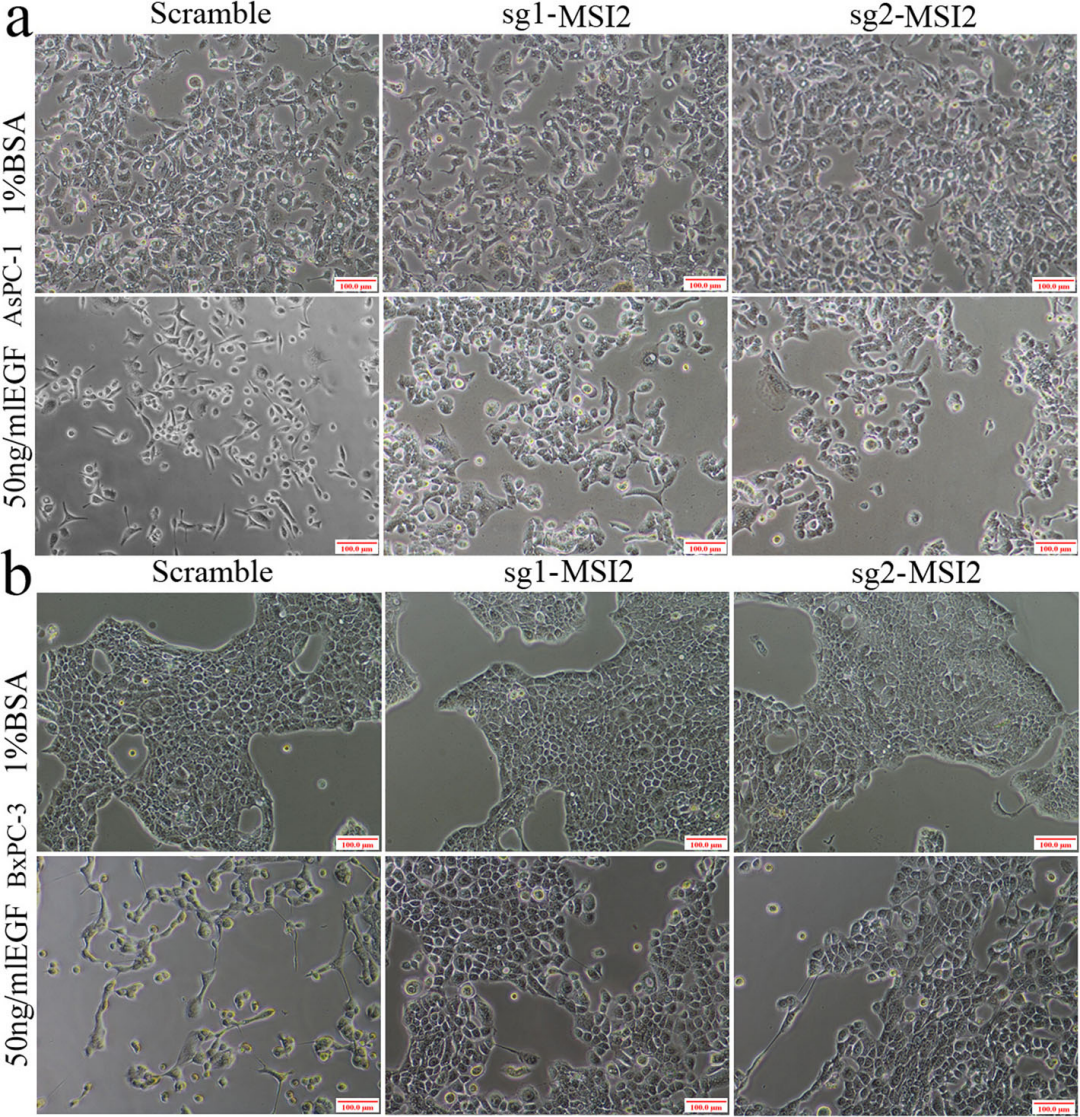
Cell morphology in sg1-MSI2, sg2-MSI2 and scramble transfected AsPC-1 and BxPC-3 cells with or without EGF (50 ng/ml) treatment. **a** and **b** Under EGF treatment, the fibroblastoid-like phenotype in AsPC-1 (**a**) and BxPC-3 (**b**) cells in scramble, sg1-MSI2 and sg2-MSI2 groups

**Fig. 3:**
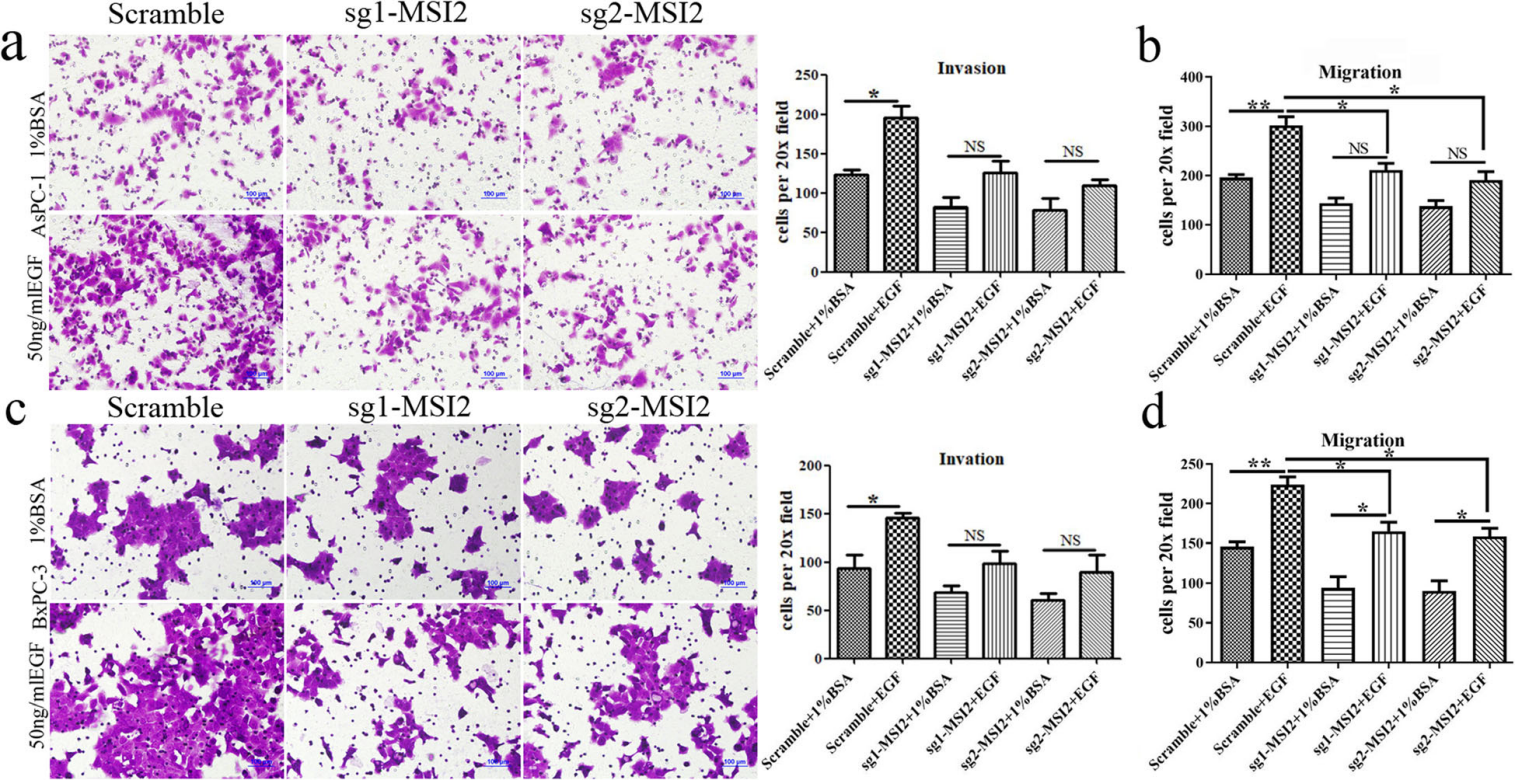
MSI2 silencing inhibited EGF-induced Cell invasion and migration in PC cells. **a** and **b** Cell invasion (**a**) and migration (**b**) in sg1-MSI2, sg2-MSI2 and scramble transfected AsPC-1 cells with or without EGF (50 ng/ml) treatment. **c** and **d** Cell invasion (**c**) and migration (**d**) in sg1-MSI2, sg2-MSI2 and scramble transfected BxPC-3 cells with or without EGF (50 ng/ml) treatment. Bars indicate ± S.E.*, *P* < 0.05; **, *P* < 0.01 compared with the control

## References

[1] Sheng W (2020). Musashi2 promotes EGF-induced EMT in pancreatic cancer via ZEB1-ERK/MAPK signaling. J Exp Clin Cancer Res.

