# Supplementary figures and images for: Musashi2 promotes EGF-induced EMT in pancreatic cancer via ZEB1-ERK/MAPK signaling
Source: J Exp Clin Cancer Res. 2020 Jan 17;39:16. doi: 10.1186/s13046-020-1521-4 (PMC6967093; doi:10.1186/s13046-020-1521-4)

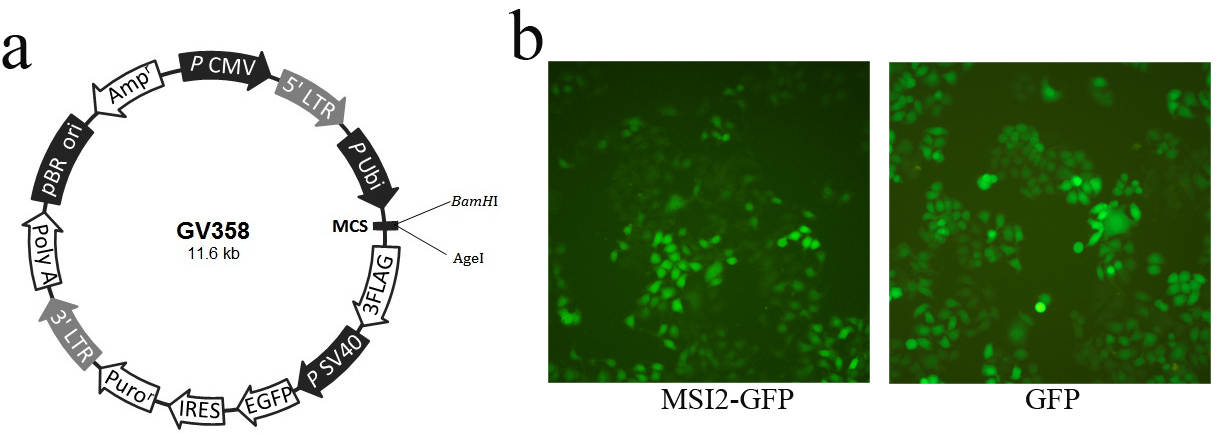

Supplement: Supplementary file 1 — Additional file 1: Figure S1 The GV358 lentivirus vector information (a) and transfected efficiency (GFP fluorescence) in MSI2 overexpressing SW1990 cells (b). [file 13046_2020_1521_MOESM1_ESM.tif]
