# Supplementary material for: Musashi2 promotes EGF-induced EMT in pancreatic cancer via ZEB1-ERK/MAPK signaling
Source: J Exp Clin Cancer Res. 2020 Jan 17;39:16. doi: 10.1186/s13046-020-1521-4 (PMC6967093; doi:10.1186/s13046-020-1521-4)
Supplement: Supplementary file 2 — Additional file 2: Table S1. The target sequences of sg1-MSI2, sg2-CRT, MSI2siRNA and corresponding scramble. [file 13046_2020_1521_MOESM2_ESM.docx]

Table S1. The target sequences of sg1-MSI2, sg2-CRT, MSI2siRNA and corresponding scramble.

| Gene | Oligo Name | Oligo Sequence |
| --- | --- | --- |
| MSI2 | sg1-MSI2 | ATCCCACTACGAAACGCTCC |
|  | sg2-MSI2 | TCCTCGTCGAGCGCAACCCA |
| sgRNA | Scramble | CGCTTCCGCGGCCCGTTCAA |
| MSI2 | siRNA | 5‘GACCCAGCAAGUGUAGAUATT 3’ |
| NC | siRNAcontrol | 5‘ UUCUCCGAACGUGUCACGUTT 3’ |
